# High-speed ultrasound imaging in dense suspensions reveals impact-activated solidification due to dynamic shear jamming

**DOI:** 10.1038/ncomms12243

**Published:** 2016-07-20

**Authors:** Endao Han, Ivo R. Peters, Heinrich M. Jaeger

**Affiliations:** 1 James Franck Institute, The University of Chicago, Chicago, Illinois 60637, USA; 2 Department of Physics, The University of Chicago, Chicago, Illinois 60637, USA; 3 Engineering and the Environment, University of Southampton, Highfield, Southampton SO17 1BJ, UK

## Abstract

A remarkable property of dense suspensions is that they can transform from liquid-like at rest to solid-like under sudden impact. Previous work showed that this impact-induced solidification involves rapidly moving jamming fronts; however, details of this process have remained unresolved. Here we use high-speed ultrasound imaging to probe non-invasively how the interior of a dense suspension responds to impact. Measuring the speed of sound we demonstrate that the solidification proceeds without a detectable increase in packing fraction, and imaging the evolving flow field we find that the shear intensity is maximized right at the jamming front. Taken together, this provides direct experimental evidence for jamming by shear, rather than densification, as driving the transformation to solid-like behaviour. On the basis of these findings we propose a new model to explain the anisotropy in the propagation speed of the fronts and delineate the onset conditions for dynamic shear jamming in suspensions.

## Abstract (web summary)

Suspensions of particles at high volume fractions are subject to discontinuous shear thickening or even turn into solid upon impact, yet the underlying mechanism remains elusive. Here, Han *et al*. follow the propagation of shear bands at jamming fronts in three dimensions and show no sign of densification.

## Graphical abstract

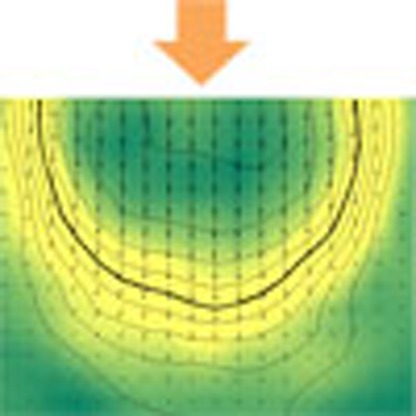

Dense suspensions are complex fluids that can exhibit strong, discontinuous shear thickening, where the viscosity jumps up orders of magnitude when a critical shear stress is exceeded^1^,^2^,^3^,^4^. Under a wide range of dynamic conditions, dense suspensions can also undergo a transformation to solid-like behaviour, for example, during sudden impact at their free surface^5^,^6^,^7^, ahead of quickly sinking objects^8^,^9^, under shear^10^ or during rapid extension^11^. Detailed investigation of the dynamics during impact has shown how such solidification is associated with a propagating front that converts fluid-like, unjammed suspension into rigidly jammed material in its wake^5^,^12^. This dynamic jamming front moves through the suspension with a speed much greater than the impactor itself.

To explain this solidification, a model was proposed^5^ that assumed the impact pushes the particles closer together until they jam. This densification scenario was based on the standard jamming phase diagram for frictionless hard particles, where entry into a jammed state requires an increase in particle packing fraction *ϕ* (ref. 13). Since the volume of particles is conserved, the front propagation speed *v*_f_ along the direction of impact is then related to the impactor speed *v*_p_ via^14^


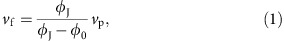


where *ϕ*_J_ is the packing fraction at which jamming occurs and *ϕ*_0_<*ϕ*_J_ is the packing fraction of the initially unjammed suspension at rest. The closer the initial packing fraction is to jamming, the faster the front will propagate, in principle diverging at *ϕ*_J_. This model shows excellent agreement with measurements of *v*_f_ in systems where the local packing fraction can change easily, such as dry granular particle layers that are being compacted snowplough-like from one end^14^.

In suspensions, the presence of an interstitial liquid makes it possible to prepare three-dimensional (3D) systems at packing fractions *ϕ* well below *ϕ*_J_ by density matching the particles to the liquid. Given that such systems can still exhibit impact-induced solidification, jamming by densification would imply significant particle packing fraction changes Δ*ϕ*=*ϕ*_J_−*ϕ*. However, unless the impact speed is so high that the liquid becomes compressible^15^, viscous drag will counteract any densification of the particle sub-phase. This calls into question the mechanism underlying equation (1), even though there is experimental evidence for the basic outcome, namely that the ratio *v*_f_/*v*_p_ increases dramatically as Δ*ϕ* approaches zero^5^,^12^.

One intriguing alternative mechanism has recently emerged with the concept of jamming by shear^3^,^16^. In this extension of frictionless, standard jamming, the presence of frictional interactions between particles makes it possible to start from initially isotropic, unjammed configurations at *ϕ*=*ϕ*_0_<*ϕ*_J_ and, without changing *ϕ*, rearrange the particles into anisotropic fragile or jammed configurations by applying shear. Shear jamming is also possible in frictionless systems, albeit over a much smaller range in Δ*ϕ* (ref. 17). So far, such shear jamming has been observed experimentally in two-dimensional (2D) dry granular systems under quasi-static conditions, where there is direct visual access to particle positions and stresses by imaging perpendicular to the 2D plane^16^. Investigating the role of shear jamming in dynamic impact-induced solidification of 3D suspensions requires the capability of non-invasively tracking the jamming fronts and the associated, quickly evolving flow field in the interior of an optically opaque system.

Here we achieve this with ultrasound. Related methods have been applied to studying dry granular materials^18^,^19^ and steady-state flow in suspensions^20^,^21^. Measuring the speed of sound *c* we obtain an upper bound on the change of packing fraction Δ*ϕ* as the suspension jams. We find that at *v*_p_<<*c*, Δ*ϕ* is much smaller than required if densification was the primary driver for impact-activated solidification. To investigate the crossover as *v*_p_ increases, we use high-speed ultrasound imaging to track the emergence of concentrated shear bands at the location of the propagating jamming fronts. In the regime of small *v*_p_, the suspension responds to stress like a fluid, and in the regime of large *v*_p_, the suspension develops solid-like characteristics, which we identify by investigating the flow fields. The invariant packing fraction and existence of shear bands provides direct evidence of dynamic shear jamming in 3D suspensions. Furthermore, access to the full flow field allows us to extract the local shear rates and identify the origin of a key, but so far unexplained, feature of the response to impact: the longitudinal front propagation speed exceeds the transverse propagation speed by a factor very close to two^12^.

## Results

### Experimental set-up and extraction of the flow field

**Figure 1:**
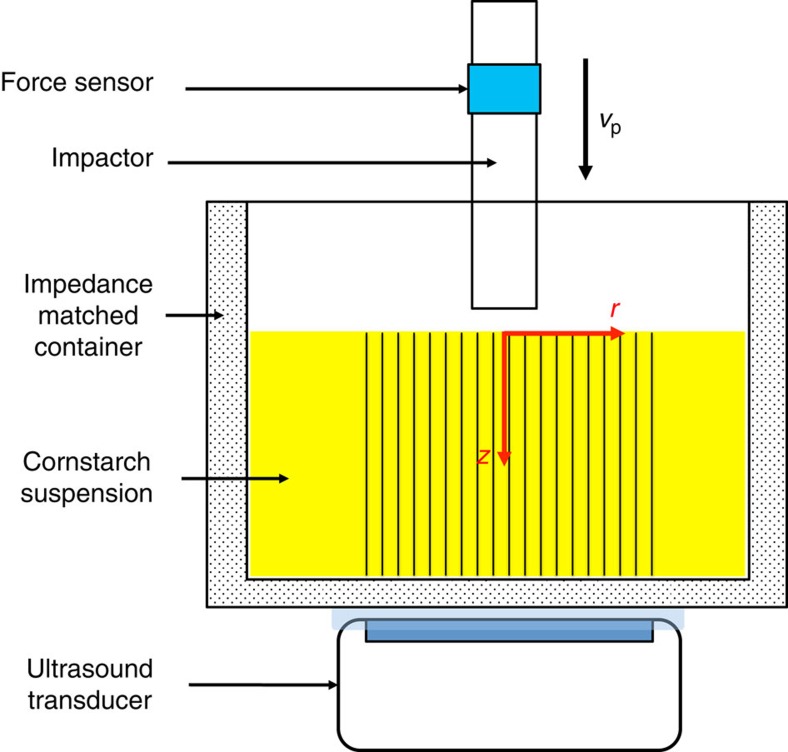
Schematic illustration of the experimental set-up. The sample container and impactor are cylindrical and concentric. The ultrasound transducer scans a vertical slice centred along the central *z* axis, providing a field of view as indicated by the striped area.

**Figure 2:**
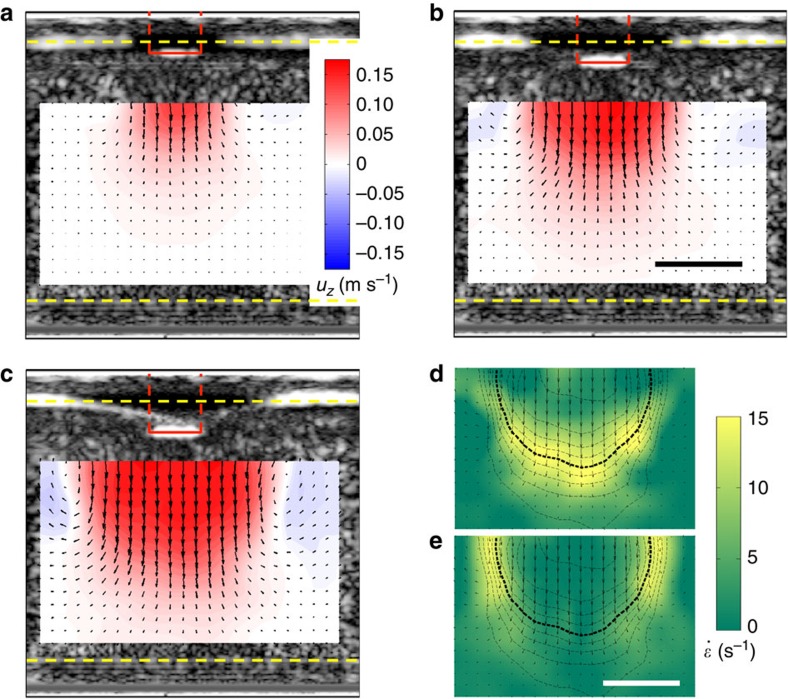
Visualization of the flow field with ultrasound. (**a**–**c**) Velocity field during an impact at time 6.0 ms (**a**) 13.2 ms (**b**) and 20.3 ms (**c**) (the impactor reached the surface when time *t*=0 ms). The images are snapshots from a high-speed ultrasound movie (shown in grey scale) with overlaid velocity field from particle image velocimetry (PIV) analysis. The colour code represents the magnitude and sign of the vertical component of the local velocity *u*_*z*_ (red corresponds to downward, blue to upward flow). Dashed yellow lines indicate the locations of the free surface of the suspension and of the bottom of the container. The impactor is outlined in red. The experimental parameters are *ϕ*=0.47, *v*_p_=175 mm s^−1^, liquid viscosity *η*_0_=4.6±0.2 mPa·s, fill depth *H*=30 mm, and impactor diameter of 6.0 mm. The black scale bar in (**b**) represents 1 cm for (**a**–**c**). (**d**,**e**) Two components of the shear rate tensor, 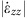 (**d**) and 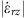 (**e**) shown for the same instant in time as the flow field in (**c**). Dashed lines are contours connecting points with the same *u*_*z*_. The thicker line indicates *u*_*z*_=*v*_p_/2, which defines the front position. Scale bar, (**d**,**e**) 1 cm. The whole process is shown in Supplementary Movie 1.

The experiments were performed with a model suspension: cornstarch particles dispersed in water–glycerol CsCl solutions. The experimental set-up is illustrated in Fig. 1. In the impact experiments the impactor was driven vertically downward with constant velocity *v*_p_ by a linear actuator. A representative flow field (*u*_*r*_, *u*_*z*_) inside the suspension during an impact is shown in Fig. 2a–c. The vertical and horizontal axes in the image correspond to the *z* and *r* directions in cylindrical coordinates. The flow field shows a jammed solid-like plug in the centre, as evidenced by the fact that all points move vertically with speed close to *v*_p_. Also evident is a strong velocity gradient around the periphery of the jammed region. To show this more explicitly, we calculate the local shear rate from the velocity field (*u*_*r*_, *u*_*z*_). Given rotational symmetry, the shear rate tensor becomes


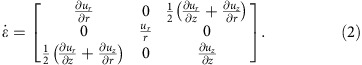


Figure 2d,e shows the two components 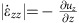 and 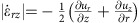. Underneath the jammed region, that is, in longitudinal direction, 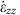 dominates. This corresponds to pure shear that compresses the suspension in the *z* direction and expands it radially. By contrast, along the sides of the jammed plug 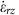 dominates, and here the main contribution arises from the term 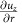. As a result, the velocity gradient is mainly perpendicular to *v*_p_. This is analogous to simple shear as seen, for example, in parallel plate setups. We will return below to the implications of having both types of shear.

### Speed of sound

**Figure 3:**
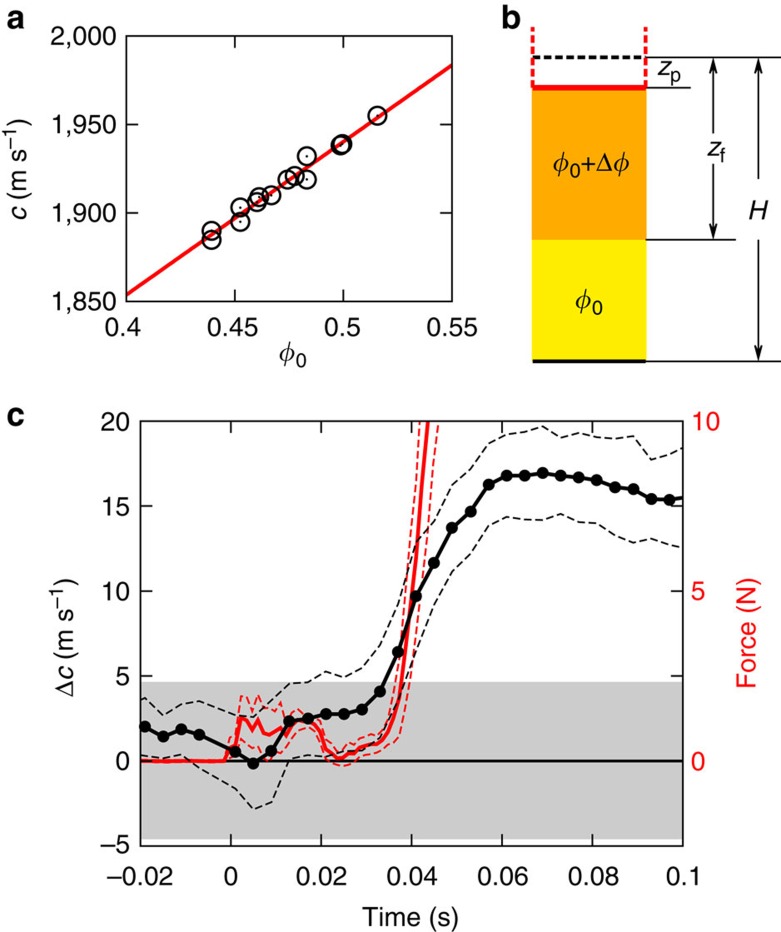
Direct measurement of packing fraction changes. (**a**) Speed of sound *c* as a function of packing fraction *ϕ*. All the data were taken with suspensions in their quiescent fluid-like state, at packing fractions well below *ϕ*_J_. From the scattering of the data points we can see the overall uncertainty of this experiment. (**b**) Sketch of the region beneath the impactor. The black dashed line represents the initial suspension surface at a fill height *H* above the bottom of the container (bold black solid line). As the impactor (outlined in red) pushes down a vertical distance *z*_p_ the front (orange region) propagates a distance *z*_f_. (**c**) Change in sound speed Δ*c* as a function of time (black trace) at *ϕ*_0_=0.48. Impact at the free suspension surface occurs at *t*=0 ms. Once the jamming front has reached the bottom of the container, the suspension below the impactor has been transformed into a solid-like material. At that point, near *t*=35 ms, the force on the impactor (red trace) rises markedly. Note that within our experimental uncertainties, the speed of sound does not increase until the shear-jammed region becomes compressed. Data are averages from seven experiments that simultaneously measured force and sound speed as functions of time. Dashed lines indicate 1 s.d. The grey region shows the uncertainty (given by 1 s.d.) in determining Δ*c* at low *v*_p_, where no solidification takes place.

In an unjammed suspension of solid particles in a Newtonian liquid, the shear modulus vanishes. In the limit that the solid particles are much smaller than the wavelength, the speed of sound is then given by 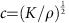, where *K* is the average bulk modulus and *ρ* the average material density of the suspension^22^,^23^. In our experiments, the particles and suspending solvent are density matched (see Methods section), but the average *K* still increases with *ϕ* since cornstarch particles have a bulk modulus larger than that of the liquid^24^. As shown in Fig. 3a, the resulting dependence of *c* on *ϕ* is, to a good approximation, linear across the regime of packing fractions probed by our experiments. This allows us to obtain Δ*ϕ* straightforwardly by detecting changes in *c* with ultrasound.

A schematic illustration of the suspension under impact is shown in Fig. 3b, indicating two regions: a jammed region (orange) directly underneath the impactor and an unjammed region (yellow) ahead of the jamming front. Our measurements provide the average speed of sound 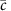 as determined from the time it takes sound pulses to traverse both regions (see Methods section). The process of transforming unjammed suspension to jammed suspension could be expected to increase the speed of sound via three possible mechanisms: (1) an increase Δ*ϕ* in packing fraction; (2) an increase in effective bulk modulus *K*; (3) the development of a finite shear modulus *G* as the suspension jams^13^. Currently, we cannot determine how much they each contribute, but we can estimate the upper limit of each individual term by assuming the other two zero.

First, we estimated the maximum possible increase in *ϕ*. Figure 3c shows the measured change in sound speed Δ*c*=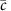
−*c*(*ϕ*_0_) during impact for a suspension prepared at *ϕ*_0_=0.48. The impactor hits the suspension surface at time *t*=0, generating a jamming front that reaches the bottom at *t*≈0.035 s. We can identify this point by the dramatic increase in force on the impactor, as established by prior investigations of quasi-2D (ref. 12) and 3D (ref. 5) systems. Until the jamming front interacts with the bottom wall Δ*c*=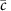
−*c*(*ϕ*_0_) is less than the measurement uncertainty of about 5 m s^−1^. Neglecting any increases in *K* and *G*, we find from Fig. 3a that our noise floor Δ*c*≈5 m s^−1^ implies Δ*ϕ*≈0.006 during the free propagation of the fronts. This means that *ϕ* could have increased to 0.49 at best. On the other hand, even at the highest packing fraction in Fig. 3a *ϕ*=0.52, the suspension can still flow when sheared slowly, implying an isotropic jamming threshold *ϕ*_J_>0.52. This means that the increase in packing fraction due to impact is much less than required for jamming by the densification model.

If instead we assume Δ*ϕ*=0 and use 
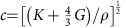
, as appropriate for solids^25^, to describe the dependence of the speed of sound on *K* and *G* within the jammed region behind the front, the same noise floor Δ*c*≈5 m s^−1^ implies that the net increase in the sum of the moduli 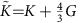 could not have been larger than 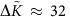 MPa. This is very small compared with the bulk modulus *K*_0_ of the quiescent suspension at *ϕ*_0_=0.48: 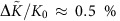.

Once the front has reached the bottom, Δ*c* increases to ≈16 m s^−1^. While this is significantly above the noise floor, it limits any packing fraction changes to Δ*ϕ*≈0.02, still less than necessary to reach *ϕ*_J_. Nevertheless, the interaction with the solid boundary increases the stress in the dynamically jammed region significantly and this drives the suspension deeper into the shear-jammed state. We can therefore expect concomitant increases in bulk and shear moduli and thus in sound speed. From the sound speed data in Fig. 3, the upper limit of the net increase in the sum of the moduli 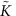 is 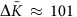 MPa.

### Propagation of fronts

**Figure 4:**
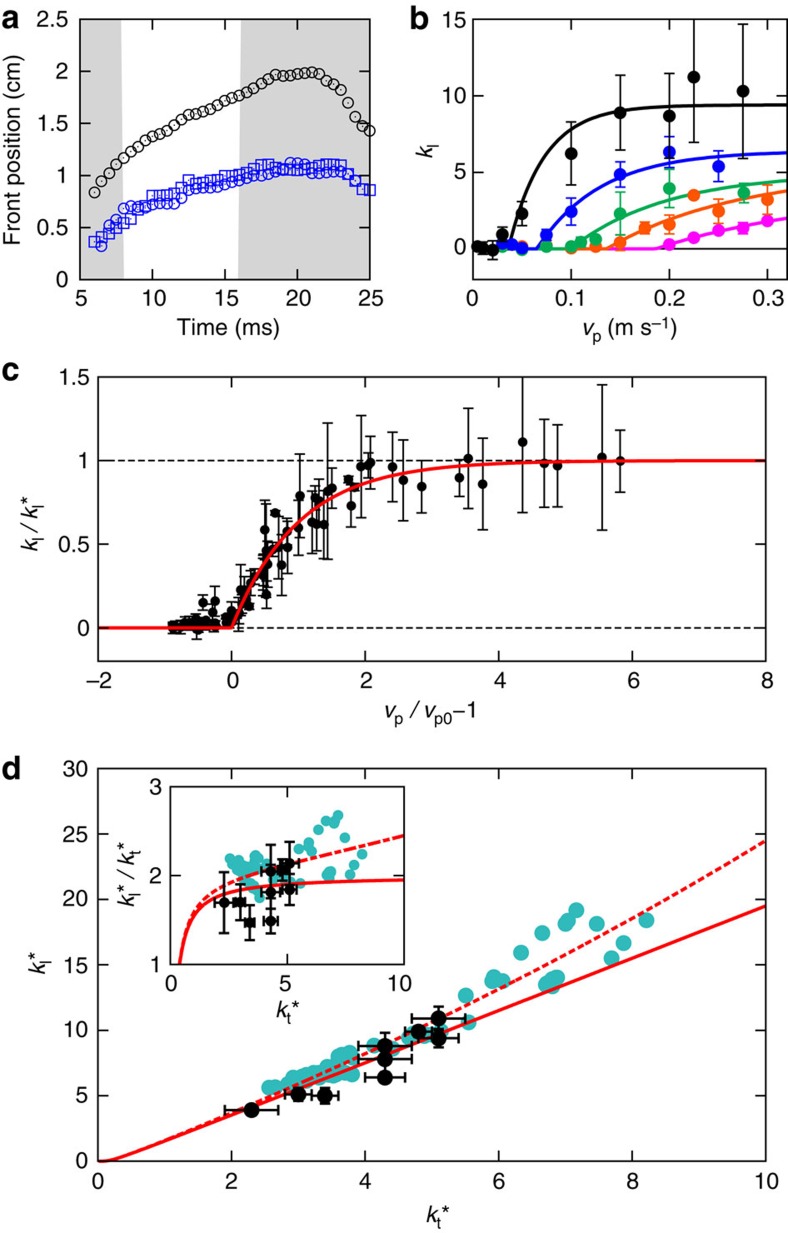
Propagation of jamming fronts. (**a**) Front position in longitudinal (black) and transverse (blue circles: right, blue squares: left) directions as functions of time. The impactor touches the suspension surface at time *t*=0 ms. The grey shaded background indicates the initial front build-up (left), and the regime where the fronts starts to interact with boundaries and slows down (right). For these data *v*_p_=200 mms^−1^, *ϕ*=0.460. (**b**) Normalized front propagation speed *k*_l_ in longitudinal direction as function of *v*_p_ for different *ϕ* (magenta: 0.439, orange: 0.453, green: 0.460, blue: 0.474, black: 0.498). All data are for suspending liquid viscosity *η*_0_=4.6±0.2 mPa·s. Error bars show the standard deviation of three measurements. The same data plotted in log-linear scale are shown in Supplementary Fig. 3. (**c**) Front speed 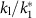 normalized by its asymptotic value as function of impactor speed *v*_p_ normalized by threshold speed *v*_p0_. Data from experiments with different *ϕ* and *η*_0_ collapse onto a master curve fit by equation (4) (solid red line). (**d**) Relationship between the asymptotic front speeds 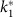 and 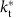 in longitudinal and transverse direction, respectively. Data from both quasi-2D (ref. 12) (turquoise) and 3D (black) impact experiments are shown. The solid red line is the prediction from equation (5). The slope approaches 2 as 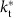 increases. The dashed red line is a modified version of the model, which includes a small strain anisotropy *δ*, here plotted using a value of *δ*=0.01. Error bars are the asymptotic s.e. from the fittings of each *k*–*v*_p_ curve with equation (4).

From the evolution of the flow fields as shown in Fig. 2, we extract the position of the moving jamming front, defining the front position as the line of points where the vertical component of the impact velocity has dropped to *v*_p_/2. In the following we focus on the points in the flow field that propagate the furthest in *z* and *r* directions, that is, on the maximum longitudinal and transverse front speeds. As shown in Fig. 4a, after an initial stage the fronts in both directions propagate essentially linearly as function of time before slowing down when they start to interact with boundaries and the incipient jammed region gets compressed by the impactor; further compression causes the motion to stop quickly (Supplementary Fig. 1). Here we investigate this linear regime, where the front propagates freely. To compare how quickly the front propagates relative to *v*_p_, we define two dimensionless front propagation factors, or normalized front speeds, *k* as


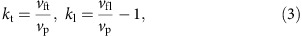


where the subscripts t and l represent transverse and longitudinal directions, respectively. The ‘−1' in *k*_l_ compensates for the vertical motion of the impactor itself.

Our experiments show that the parameters that affect *k*_l_ and *k*_t_ include *ϕ*, *v*_p_ and the suspending liquid's viscosity *η*_0_. For a suspension with given *η*_0_ and *ϕ*_0_ that is impacted very slowly, the response is fluid like and both *k*_t_ and *k*_l_ are close to zero. However, beyond a threshold value *v*_p0_ jamming fronts start to appear. Their normalized speeds initially increase quickly with impactor speed *v*_p_ but eventually asymptote to a fixed k*. The relation between *k*_l_ and *v*_p_ in suspensions with the same *η*_0_ but different *ϕ*_0_ is shown in Fig. 4b; the behaviour of *k*_t_ is similar. As *ϕ* increases, the curves shift towards lower *v*_p0_ and higher k*. For comparison, in suspensions with the same *ϕ*_0_, larger solvent viscosity *η*_0_ leads to lower threshold *v*_p0_ (Supplementary Fig. 2). To extract k* and *v*_p0_ we fit the data to


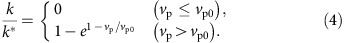


Equation (4) is not derived but a phenomenological relation that captures the key aspects of the data discussed above: (1) *k*=0 at small *v*_p_; (2) *k* increases when *v*_p_>*v*_p0_; (3) *k* approaches k* as *v*_p_→∞. Plotting the data in terms of normalized variables *k*/k* and *v*_p_/*v*_p0_ scales out the dependencies on *ϕ*_0_ and *η*_0_. The resulting data collapse for the longitudinal front speed ratio 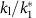 is plotted in Fig. 4c. A similar result is obtained for the transverse speed ratio 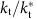.

To quantify the anisotropy in front propagation in longitudinal and transverse directions, we plot 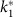 versus 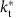 in Fig. 4d, using the data from experiments varying *ϕ* (larger packing fraction increases both 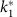 and 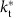) and *η*_0_. To a good approximation, all the data follow 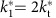. Comparison of our data with the data obtained for quasi-2D suspensions^12^ shows excellent agreement as well, except that for higher *k*_t_ the ratio *k*_l_/*k*_t_ slightly exceeds 2.

## Discussion

Our data in Fig. 3c demonstrate that impact-activated jamming of dense suspensions proceeds without significant increase of *ϕ*, and certainly without increasing *ϕ* to values close to *ϕ*_J_. This rules out earlier explanations based on entering the jammed state via densification of the particle sub-phase^5^,^14^. Instead, analysis of the flow field shows that the jamming fronts initiated by the impact coincide with the location of the maxima in local shear rate (Fig. 2d,e). Altogether, these two findings provide strong evidence for dynamic shear jamming: the impact triggers propagating fronts that locally create sufficient shear to reorganize particles into (anisotropic) jammed configurations without changing the average packing fraction. There are several implications of the shear jamming scenario for suspensions and several differences from dry granular systems, both of which we discuss next.

To start, we examine the stress. In a dry granular system, stress is sustained only via direct contact between particles. By contrast, in a dense suspension stress can also be transmitted without contact via lubrication forces. Thus, while in dry granular systems there is only a single characteristic stress scale for entry into the shear-jammed regime^16^, for a suspension the situation can be more complex. A number of theoretical models^3^, simulations^2^,^26^ and experiments^27^,^28^,^29^ have recently suggested that lubrication breaks down and particles start to experience frictional interactions beyond a local stress threshold *σ*_1_. Thus, for stress levels below *σ*_1_ the suspension behaves fluid-like, while above *σ*_1_ the system can be thought to behave more like a frictional granular system, that is, enter a fragile regime before crossing over into the shear-jammed regime at a second characteristic stress level *σ*_2_ (ref. 16). Within this picture, we associate the transition at *v*_p0_ with the situation where the stress levels at the leading edge of the jamming front have reached *σ*_1_ and are large enough for frictional interactions to occur. Thus, when *v*_p_<*v*_p0_ the suspension is in the lubrication regime, but when *v*_p_>*v*_p0_ it transitions into a fragile state with behaviour intermediate between solid and fluid^16^,^30^,^31^, as frictional contacts start percolating through the system to form a load-bearing network, eventually reaching a shear-jammed state as *v*_p_ increases further. We point out that a non-zero shear modulus is not strictly necessary for the front to propagate (with *k*>0). The front will propagate as long as it transforms the initially liquid-like suspension into a state with sufficiently large viscosity. However, we know that this state behaves solid-like, while interacting with a system boundary^5^,^6^,^10^,^12^. Thus, how the shear modulus evolves in the region behind the jamming front before a system-spanning jammed state has been established remains an open question.

The stress-based argument also provides an explanation of the relaxation or ‘melting' of the jammed region when the impact stops. During front propagation the stress inside the jammed region is sustained by the inertia of the suspension in the shear zone ahead of the jammed region. When the motion of the impactor stops, the shear zone disappears and the stress applied on the boundary of the jammed suspension falls below *σ*_1_, insufficient to sustain frictional interactions between particles and therefore any network of force chains that could generate a yield stress and support a load. As a result, the suspension returns to the lubrication regime.

However, while necessary, the existence of threshold stress levels is not sufficient to explain the asymptotic front speed k* at high *v*_p_ and the seemingly universal anisotropy in front propagation, expressed by the ratio 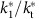
≈2. Particles also need to move out of an initial uniform isotropic distribution and reorganize under shear into anisotropic structures (force chains) that can support the stress. Such reorganization requires a minimum shear strain *ɛ*_c_ to engage neighbouring particle layers. As a result, shear jamming happens only when stress and strain both reach their threshold values. In a quasi-static granular system^16^,^17^ the threshold strain only matters when the shear-jammed state is prepared or when the shear is reversed. In the dynamic system considered here, the front continues to propagate into unperturbed suspension, and therefore, the front advances by applying strain *ɛ*_c_ locally during the whole process of front propagation.

For dense suspensions in the high *v*_p_ regime, where the stress threshold is clearly exceeded, we can show that k* is governed by *ɛ*_c_ and that, in fact, the front speed anisotropy is a direct consequence of the existence of a strain threshold. As described above, the suspension experiences pure shear in the longitudinal direction and simple shear in the transverse direction. In 2D, we can directly compare the two types of shear using the positive eigenvalues of the shear rate tensors, treating the propagation of the front in the longitudinal and transverse directions as two effectively 1D problems. We now assume that a suspension element jams when the shear strain it experiences reaches *ɛ*_c_, irrespective of propagation direction. This leads to the following relations between 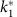, 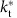 and *ɛ*_c_ (see Methods section):


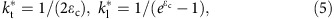


and


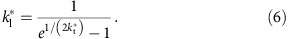


Equation (6) is plotted in Fig. 4d. For small *ɛ*_c_ we find from equation (5) that 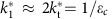. In other words, the anisotropy ratio of 2 in the normalized front speeds originates from the factor 1/2 in the non-diagonal terms of the shear rate tensor, which in turn arises because simple shear can be decomposed into a combination of pure shear and solid body rotation. In 3D, it is not possible to quantify the effects of pure shear and simple shear via the same approach (see Methods section). However, one possible solution is to use the ‘strain intensity' 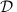 suggested by Ramsay and Huber^32^, which provides a scalar measure of the relative strength of the two types of shear. As we show in the Methods section, this leads to a ratio 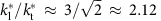, very close to the value for the 2D case. Therefore, an anisotropy ratio ≈2 agrees very well with the experimental data for both quasi-2D (ref. 12) and 3D systems within our measurement precision.

With increasing packing fraction *ϕ* the average distance between particles decreases and we expect the strain threshold *ɛ*_c_ to decrease as well, which agrees qualitatively with the measurements of *ɛ*_c_ in dry granular systems^16^. Via equation (5) this explains the increase in *k* with *ϕ* seen in Fig. 4b: since it takes less strain to reorganize the particles into a shear-jammed network the front will propagate faster for given impactor velocity. We point out that equation (1), which formalizes such relationship between packing fraction and front speed, appears to capture the overall trend qualitatively. However, this seems fortuitous, since equation (1) was based on the assumption that the moving front significantly increases the packing fraction, in fact driving it up all the way to *ϕ*_J_, which we now can rule out in our system. In addition, equation (1) cannot predict the observed propagation anisotropy. One of the outstanding tasks therefore is to develop a model for k*(*ϕ*) that is based on jamming by shear rather than densification.

An interesting aspect of the data in Fig. 4d is the deviation from the anisotropy ratio ≈2 at large k* values or, equivalently, large packing densities. This is most apparent in the data available for the quasi-2D system and it indicates that the longitudinal speed becomes faster. We speculate that this may be connected to a breakdown of the assumption of an isotropic threshold *ɛ*_c_. For example, if the impact were to introduce a small amount of compression of the particle sub-phase in longitudinal direction, *ɛ*_c_ would be reduced in that direction. This effect would become increasingly significant at large ϕ. We can model this by introducing a correction *δ* so that


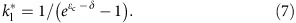


Using 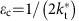 and *δ*≈0.01 we can reproduce the trend in Fig. 4d well (dotted red line). However, we point out that this is just the simplest way to account for the trend in the data and there might be other reasons for the deviation.

Taken together, these results provide important insights into the mechanism responsible for impact-induced solidification of dense suspensions. The finding that the packing fraction does not increase measurably during impact, together with the observation of strong shear at the leading edge of the propagating solidification fronts, rules out jamming via densification as the dominant mechanism and points to jamming by shear (densification is likely to play a significant role at much larger impact velocities, when the interstitial liquid's compressibility can no longer be neglected^15^). In dense suspensions this introduces a new stress scale or, equivalently, an impact velocity threshold, which we associate with the breakdown of lubrication films between particles and the onset of frictional interactions^2^,^3^,^27^,^28^,^29^. Behind the front, these frictional interactions create a dynamically shear-jammed region (corresponding to fragile and/or shear-jammed states in refs 3, 16). Further support for the shear-jamming scenario comes from the observation of anisotropic front propagation, where we can relate the fact that the fronts propagate longitudinally twice as fast as in transverse direction to an equivalent factor in the ability to transmit shear strain. We point out that for dynamic shear jamming, both shear stress and strain need to exceed threshold values, and the critical shear strain determines the front propagation speed.

## Methods

### Experimental set-up

The ultrasound measurements and imaging were performed with a Verasonics Vantage 128 system. The sample container was 3D-printed from ultraviolet-cured resin (‘Vero White Plus', Objet Geometries Inc.) whose acoustic impedance matched the suspensions we studied. The inner diameter of the container was 100 mm and the typical depth *H* of the suspension was 25 to 35 mm. This insured that the front reached the bottom before it interacted with the side wall. The impactor was a cylinder of diameter of 6 or 10 mm, driven by a computer controlled linear actuator (SCN5, Dyadic Systems) and equipped with a force sensor (DLC101, Omega). The ultrasound transducer (Philips L7-4; 128 independent elements; total width of 38 mm) contacted the bottom of the container through a thin layer of ultrasound gel. The ultrasound system was triggered as the impactor approached the surface; images were taken at a frame rate of 10 to 10,000 frames per second, adjusted according to *v*_p_. The spatial resolution of the ultrasound was limited by the wavelength, about 0.4 mm in our experiments.

### Suspensions

The cornstarch (Ingredion) was stored in the lab environment at 22.5±0.5 °C and 51±2% relative humidity. Individual particles had a diameter of 5–30 μm (refs 5, 33). The suspending solvent was a solution of CsCl, glycerol and water. Its viscosity *η*_0_ was adjusted by the mass ratio of glycerol and water, and its density was matched to that of the cornstarch particles by adjusting the mass ratio of CsCl. The density of the particles was *ρ*_cs_=(1.63±0.01) × 10^3^ kg m^−3^ as measured by density matching. To calculate the packing fraction *ϕ*, an accurate determination of the volume occupied by particles and interstitial liquid is required. For cornstarch this is difficult because the particles are porous and they already contain some moisture before they are dispersed in the suspending solvent. Therefore, often a value *ϕ*_m_ based on the mass fractions before mixing is quoted^4^,^5^,^7^, which is proportional to *ϕ*. For example, in Fig. 4b, the *ϕ*_m_ values were 0.390, 0.402, 0.409, 0.425 and 0.444. To obtain the actual packing fraction, we account for a small fraction *α* of suspending liquid that is wicked up by the porous particles and write *ϕ*=(1+*α*)*ϕ*_v_, where *ϕ*_v_ is the material volume packing fraction^34^,^35^. When calculating *ϕ*_v_, we considered the moisture in the cornstarch particles. We assume the moisture is pure water and its mass ratio in cornstarch is *β* in our lab environment. This leads to


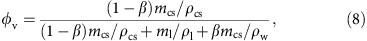


where *m*_cs_ is the mass of cornstarch particles, *m*_l_ is the mass of the solvent liquid, *ρ*_l_ is the density of the solvent and *ρ*_w_ is the density of pure water. In this paper we use *α*=0.3 and *β*=0.11 by estimation. Changing of these numbers will not affect the conclusions we make.

Since air bubbles mixed in the suspension scatter ultrasound signals significantly, the suspensions were debubbled before using. To keep *ϕ* fixed during debubbling, we filled the suspensions into sealed syringes and then lowered the pressure by pulling the plungers. The syringe walls were tapped gently to help bubbles separate from the suspension. After debubbling a small amount of suspension was used to measure the speed of sound *c* as required for image reconstruction. For imaging the flow field, a small amount of air bubbles were added back to the debubbled suspensions to act as tracer particles. This was done by slowly stirring the suspension, then tilting and slowly rotating the container till the bubbles were uniformly distributed. We verified that the amount of bubbles did not suppress the penetration of the ultrasound in the suspensions and produced a negligible change in the speed of sound. We also determined that the effect of the bubbles on the suspension viscosity is limited (Supplementary Fig. 4). Between successive impact experiments the suspension was relaxed by gently shaking and rotating the container.

### Data acquisition and analysis

Once triggered, the ultrasound system made several hundred acquisitions consecutively. In one acquisition each of the 128 transducer elements transmitted the same ultrasonic pulse at the same time and received an individual reflected time series. The pulse was a 5-MHz sinusoidal wave modulated by a Gaussian profile (Gaussian envelope) for a pulse length of 6 periods. From the time series received by the transducer and using the previously measured speed of sound *c* we reconstructed B-mode images (using brightness to represent the echo signal amplitude)^25^ that captured the positions of the tracer particles (air bubbles) in the suspension. Given our finding that *c* does not change measurably during the impact, the image reconstruction process does not need to account for spatial or temporal variations in *c*. By tracking the tracer bubble displacements with a particle imaging velocimetry (PIV) algorithm, we obtained a 2D flow field from within the bulk of the suspension.

### Change of packing fraction measurements

The experimental set-up was identical to the one illustrated in Fig. 1 and a schematic illustration is shown in Fig. 3b. The impactor started from the surface of the suspension and pushed down a distance *z*_p_. The position of the impactor was measured with a high-speed camera (Phantom V9, Vision Research). The ultrasound measured the time of flight *T* of the signal transmitted from the bottom, reflected by the impactor and sent back to the bottom. Thus the average speed of sound 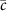 along this path is


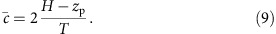


We started with experiments at a low *v*_p_ (5 mm· s^−1^) to measure the speed of sound in the liquid-like, unjammed suspension, where 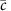
=*c*(*ϕ*_0_). Define *T*_0_ as the initial time of flight when *z*_p_=0 mm, we have *H*=*c*(*ϕ*_0_)*T*_0_/2, and from this


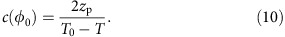


The initial packing fraction *ϕ*_0_ in these experiments was 0.48. The liquid was a mixture of 44.3% CsCl, 27.8% glycerol and 27.8% water by mass, with *η*_0_=4.6 mPa·s and *ρ*=1.63 × 10^3^ kg·m^−3^. From six measurements we obtain *c*(*ϕ*_0_)=1939.2±4.6 m s^−1^ and *H*=*c*(*ϕ*_0_)*T*_0_/2=34.1±0.1 mm.

For the high *v*_p_ (200 mm s^−1^) experiments we used the value of *H* measured above and equation (9) to calculate 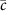. The time of flight now becomes


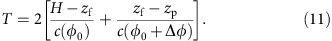


For Δ*ϕ*=0, *T*_Δ*ϕ*=0_=2(*H*−*z*_p_)/*c* (*ϕ*_0_). If Δ*ϕ*>0, there will be a difference between *T* and *T*_Δ*ϕ*=0_, and the difference becomes increasingly large as *z*_f_ increases, which leads to an increase in 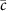 according to equation (9).

### Derivation of equation 5 in 2D

For an idealized 2D system we define a Cartesian coordinate with *x* axis in the transverse and *y* axis in the longitudinal direction. To obtain the relation between the strain threshold *ɛ*_c_ and the normalized front speeds *k* we consider how much shear strain a suspension element experiences when it accelerates from *u*_*y*_=0 to *u*_*y*_=*v*_p_. We consider the propagation in the transverse and longitudinal directions separately as two quasi-1D problems. Exemplary sketches of the velocity profiles are provided in Supplementary Fig. 5. The experimental data did not show a significant change in front width, so here we assume the shape of the front does not change during propagation. In this case the velocity profiles can be expressed as


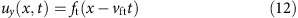


in the transverse direction and


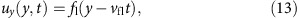


in the longitudinal direction. In both the equations *t* is time, *v*_ft_ and *v*_fl_ are front propagation speeds. *f*_t_(*X*) and *f*_l_(*X*) are functions that satisfy *f*_t_=*f*_l_=*v*_p_ as *X*→−∞ and *f*_t_=*f*_l_=0 as *X*→+∞.

On either side of the impactor the front propagates in transverse direction and the front speed *v*_ft_=*k*_t_*v*_p_, while the suspension itself is sheared longitudinally by the advancing front. The acceleration of a suspension element is then


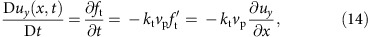


where *D*/*Dt* is the material derivative and 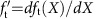. Below the impactor there are two differences: one is that the suspension element now moves along the propagation direction of the front and the other is that *v*_fl_=(*k*_l_+1)*v*_p_ as defined in equation (3). The acceleration then becomes


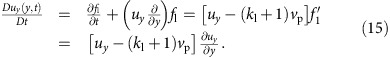


Now we look at the relation between the local shear rate 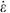 and the velocity gradient. In general, for an incompressible 2D fluid the shear rate tensor is


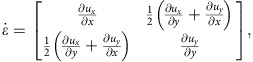


where 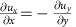. From experimental observation we have 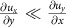. For the transverse direction, where simple shear dominates, the diagonal terms vanish and the shear rate tensor becomes


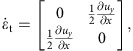


while for pure shear in the longitudinal direction the off-diagonal terms vanish and we have


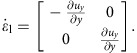


In either case the matrix has two eigenvalues with the same magnitude but opposite sign, and the eigenvalues represent the shear rate on the principal axes. Thus, we can represent the shear intensities by the tensors' positive eigenvalues: 
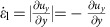
 and 
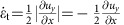
.

Using the velocity gradient, we relate the local shear rate with the acceleration of the element:


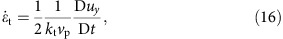



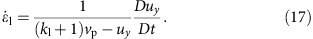


Consequently, the total shear strain *ɛ* a suspension element experiences before jamming is:


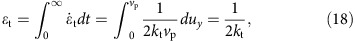


and


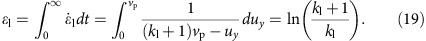


Equation (19) gives *ɛ*_l_≈1/*k*_l_ for *k*_1_>>1. If we assume the strain threshold to jamming *ɛ*_c_ is isotropic, then *k*_t_=1/(2*ɛ*_c_) and 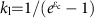.

### Relation between *k*_l_ and *k*_t_ in 3D

In 3D, the shear rate tensor is shown in equation (2). In the longitudinal direction, pure shear dominates and the shear rate tensor is


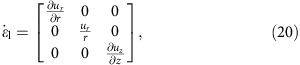


where 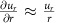 and 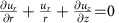. In the transverse direction simple shear dominates. This gives


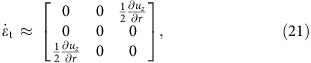


where we have used 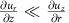. Note that though the system is 3D simple shear only operates in the *rz* plane while leaving the azimuthal direction invariant. The eigenvalues become 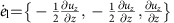 and 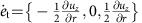. Unlike the 2D case, we cannot simply use a positive eigenvalue to represent the shear intensity. However, we can define infinitesimal strains *e*_*i*_ (*i*=1, 2, 3) along the three principal axes and rank-order them according to *e*_1_>*e*_2_>*e*_3_. Following the definition given in ref. 32, the ‘strain intensity' 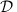 is


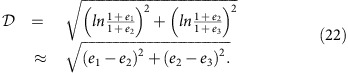


For pure shear in the longitudinal direction *e*_1_=*e*_2_=−*e*_3_/2 and 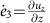, so 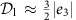, which leads to 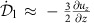. For simple shear in the transverse direction *e*_1_=−*e*_3_, *e*_2_=0 and 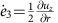. This leads to 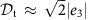, and therefore 
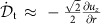
. Following the procedure for the 2D case we have


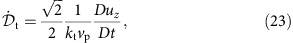



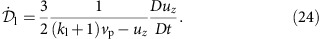


Integration then leads to


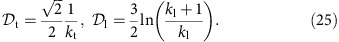


Now we again assume that the system shear-jams when 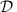 reaches a threshold strain value 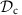, independent of the type of shear it experiences. From this we find


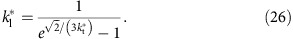


and 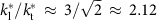 for large *k*.

### Data availability

The data that support the findings of this study are available from the corresponding author on request.

## Additional information

**How to cite this article:** Han, E. *et al*. High-speed ultrasound imaging in dense suspensions reveals impact-activated solidification due to dynamic shear jamming. *Nat. Commun.* 7:12243 doi: 10.1038/ncomms12243 (2016).

## Supplementary Material

Supplementary Information Supplementary Figures 1-5

Supplementary Movie 1 Visualization of the flow field with ultrasound. The movie shows the velocity field of the flow and the shear rate distribution in the suspension under impact.
