# Supplementary material for: High-speed ultrasound imaging in dense suspensions reveals impact-activated solidification due to dynamic shear jamming
Source: Nat Commun. 2016 Jul 20;7:12243. doi: 10.1038/ncomms12243 (PMC4961793; doi:10.1038/ncomms12243)
Supplement: Supplementary Information — Supplementary Figures 1-5 [file ncomms12243-s1.pdf]

Supplementary Information

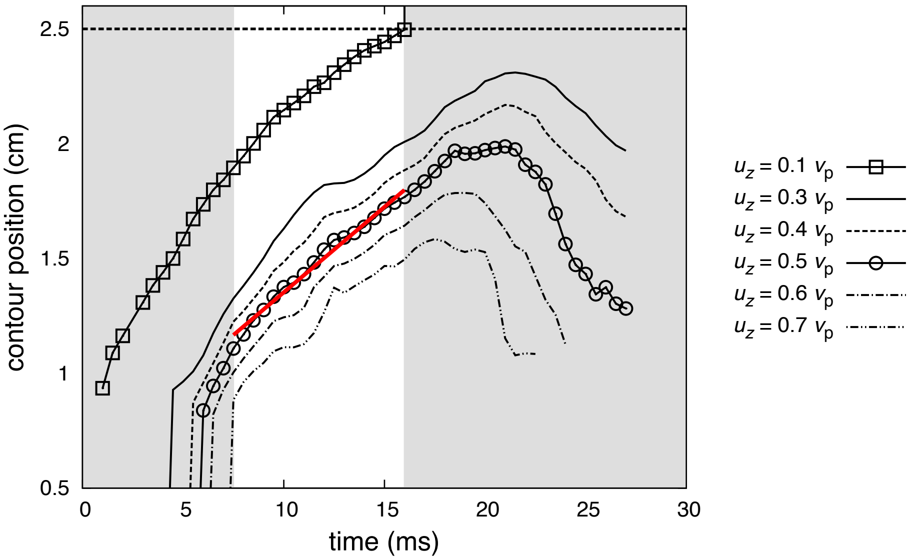

Supplementary Figure 1 | Propagation of contours at different u_z in the longitudinal direction. In the main text we define the position of the front as the position of the contour at u_z = 0.5v_p. Here we look at the positions of some other contours at different u_z. The data shown here and in Fig. 3b are from the same experiment. Each black curve shows the z position of the points that propagate the furthest in the longitudinal direction on the corresponding contour. At the very beginning of the impact the front propagates faster because it has not reached a steady state. We define the end of linear propagation regime as the point in time where the u_z = v_p/10 contour reaches the black dashed line that is set slightly above the bottom of the container. The red line shows the linear fitting.

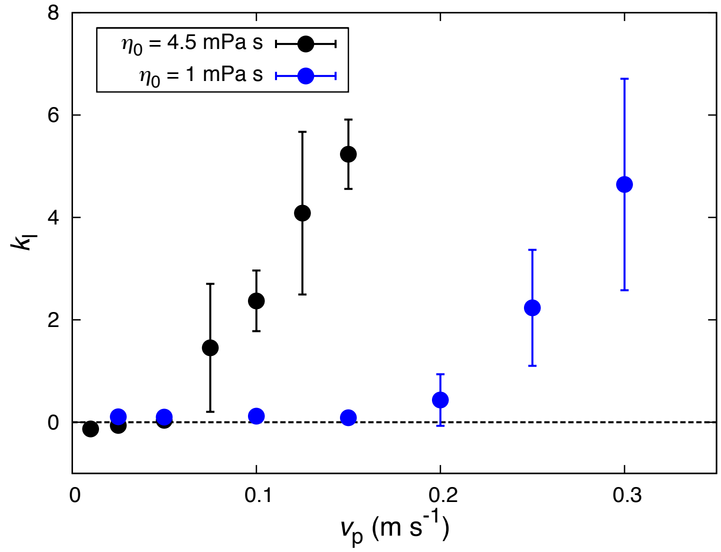

Supplementary Figure 2 | Longitudinal propagation factor k_l as functions of v_p at different liquid viscosities η_0. We measured k_l as function of v_p in suspensions at the same packing fraction $\phi = 0.48$ but different η_0. Error bars show one standard deviation, based on three measurements. The data show that as η_0 increases, the critical velocity v^* decreases.

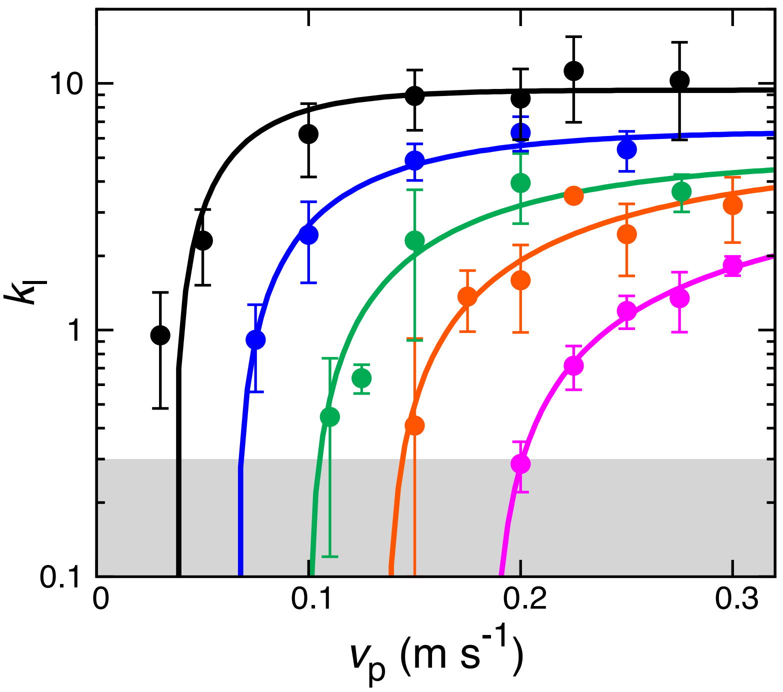

Supplementary Figure 3 | Relation between k_l and v_p plotted in log-linear scale. The data are identical to those shown in Fig. 3b, but here only the data points at $v_p > v^*$ are plotted. The grey region indicates the estimated noise level.

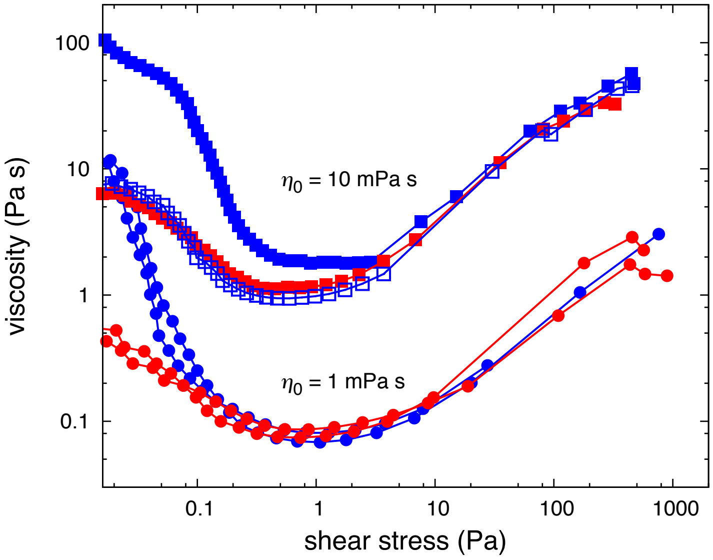

Supplementary Figure 4 | Viscosity of suspensions with and without debubbling. We measured the viscosity of cornstarch suspensions before and after debubbling with a parallel plate setup (Anton Paar rheometer, model MCR 301). ϕ = 0.47 for all the data. Blue circles: η_0 = 1 mPa s, without debubbling; red circles: 1 mPa s, debubbled; solid blue rectangles: η_0 = 10 mPa s, without debubbling, measured immediately after the sample was made; hollowed blue rectangles: η_0 = 10 mPa s, without debubbling, measured approximately two hours after the sample was made; solid red rectangles: η_0 = 10 mPa s, debubbled. The data show a large difference between the suspensions with and without debubbling at low shear stress. When the stress is high the viscosities are very similar. For the suspension without debubbling but left undisturbed for a while, the response is basically indistinguishable from the debubbled case, and the ultrasound images show that the intensity of scattering is intermediate between the debubbled ones and the freshly made suspensions. Presumably after two hours the volume fraction of air decreases and the average size of the bubbles has become smaller.

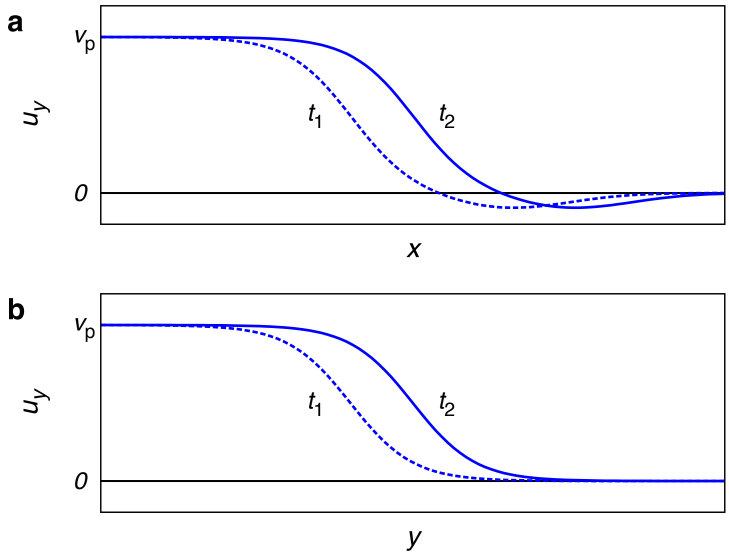

Supplementary Figure 5 | Schematic illustrations of the front profiles along the transverse (x) and longitudinal (y) directions in 2D. (a) Front profiles in the transverse direction at two times t_1 and t_2 ($t_2 > t_1$). In the jammed region $u_y = v_\mathrm{p}$ and in the shear zone it drops quickly to zero. It goes slightly negative at larger x because of the circulation outside of the jammed region, which can be seen in Fig. 1b. u_p returns back to zero far away from the impactor. (b) Front profiles in the longitudinal direction at t_1 and t_2. Both front profiles $f_\mathrm{t}(x - v_\mathrm{kt}t)$ and $f_\mathrm{l}(y - v_\mathrm{kl}t)$ approach v_p when x or $y \to 0$ and approach 0 when x or $y \to +\infty$.
